# Unraveling brucellosis: advances in pathogenesis, diagnostic strategies, therapeutic innovations, and public health perspectives

**DOI:** 10.3389/fmed.2025.1629008

**Published:** 2025-10-08

**Authors:** Abdulaziz M. Almuzaini, Ayman Elbehiry

**Affiliations:** ^1^Department of Veterinary Preventive Medicine, College of Veterinary Medicine, Qassim University, Buraydah, Saudi Arabia; ^2^Department of Public Health, College of Applied Medical Sciences, Qassim University, Buraydah, Saudi Arabia

**Keywords:** brucellosis, pathogenesis, diagnosis, treatment regimens, public health

## Abstract

Brucellosis remains one of the most impactful zoonotic diseases worldwide, posing major socioeconomic and public health challenges, particularly in low- and middle-income countries. This review presents recent progress in understanding the pathogenesis of *Brucella* species, emphasizing the role of key adhesins—*SP29, SP41, BigA, BigB, BamA*, B*maB, BmaC, Bp26, BtaF*, and *BtaE*—in host-pathogen interactions that drive adhesion, invasion, and immune evasion. We also critically assess current diagnostic approaches, including conventional culture techniques, serological assays, and emerging molecular platforms, which offer improved sensitivity and specificity. Current treatment regimens involve extended antibiotic combinations—typically doxycycline with rifampin or streptomycin—and may include surgical intervention in complicated cases. Additionally, the integration of nanotechnology-based drug delivery and traditional Chinese medicine offers promising adjunctive therapies. Although several animal vaccines exist, no approved vaccine is currently available for human use. Novel vaccine platforms, including live vectors, DNA subunits, and nanoparticle-based formulations, are under development. Finally, we address the disease's broad socioeconomic impact—ranging from livestock losses to healthcare burdens—and highlight ongoing challenges, such as diagnostic limitations, antimicrobial resistance, underreporting, and barriers to vaccine development. A One Health approach, alongside translational research and integrated surveillance, is vital to advancing prevention and control strategies for this neglected zoonosis.

## 1 Introduction

Brucellosis is a globally significant zoonotic disease that continues to pose serious public health, veterinary, and socioeconomic challenges. It is caused by gram-negative, facultative intracellular coccobacilli of the genus *Brucella*, which infect a wide range of domestic and wild animals and can be transmitted to humans through direct contact with infected animals or the consumption of contaminated animal products, especially unpasteurized dairy products (1, 2). In many developing regions, brucellosis remains endemic, placing considerable burdens on public health systems and agricultural economies due to decreased productivity, increased abortion rates in livestock, and chronic illness in humans (3).

The genus *Brucella* includes several species with varying host preferences and pathogenic potential. Among these, *Brucella melitensis* (*B. melitensis*), *Brucella abortus* (*B. abortus*), *Brucella suis* (*B. suis*), and *Brucella canis* (*B. canis*) are of particular concern to human health. *B. melitensis* is considered the most virulent and is most frequently associated with human brucellosis, particularly in endemic regions such as the Middle East, the Mediterranean basin, parts of Asia, and Latin America (4, 5). The genus includes three highly pathogenic species—*B. abortus, B. melitensis*, and *B. suis*—which primarily infect livestock. Among these, *B. melitensis* is the most virulent in humans and is responsible for the majority of severe brucellosis cases worldwide (3, 6). Most cases occur in the Mediterranean, Central Asia, the Middle East, South Asia, North Africa, and Latin America (7, 8).

*Brucella* relies on cyclic glucans, the VirB type IV secretion system, and modified lipopolysaccharides (LPSs) for invasion and replication, as it lacks conventional virulence factors (9, 10). Compared with other gram-negative bacteria, its LPS elicits a limited immune response (6, 11). Additionally, genomic islands and outer membrane proteins (e.g., *BacA, SagA, BmaC, BetB, BtaE, MucR*) play pivotal roles in pathogenicity (1).

The clinical manifestations of brucellosis range from asymptomatic to severe and include prolonged fever, night sweats, joint pain, fatigue, weight loss, abdominal discomfort, and hepatosplenomegaly, with complications such as endocarditis and neurological disorders (12–14). Neurobrucellosis is a rare but severe complication of human brucellosis that presents significant diagnostic and therapeutic challenges. Its clinical spectrum includes neurological and psychiatric manifestations such as meningitis, meningoencephalitis, myelitis, psychosis, personality changes, and persistent fatigue-like syndromes. These symptoms often mimic other infectious or autoimmune disorders, leading to frequent misdiagnosis or delayed treatment (15, 16). Effective treatment typically requires prolonged, multi-agent therapy—commonly combining ceftriaxone, doxycycline, and rifampin—with durations of several months (17, 18). Relapse remains a concern even after extended courses, highlighting the necessity for sustained clinical vigilance and follow-up (19). Given the condition's varied presentations and potential for chronic morbidity, clinicians—especially in endemic or high-risk occupational settings—must maintain a high index of suspicion.

Various *Brucella* species infect animals such as cattle, sheep, goats, and dogs (20, 21), with human infections commonly arising from contact with infected livestock or the consumption of unpasteurized dairy products (7, 22). Transmission occurs primarily through the ingestion of raw dairy products, contact with infected tissues, or inhalation of airborne particles (14), whereas human-to-human transmission is rare (23).

Conjunctival exposure also represents an important transmission route, particularly when infectious particles contaminate the eyes of individuals assisting with animal parturition. Several studies have documented that mucosal exposure during birthing practices and veterinary procedures significantly increases the risk of human infection (24, 25). In addition, nosocomial transmission has been reported, placing healthcare and laboratory workers at elevated risk of accidental infection through handling of clinical specimens or cultures. Hospital-based outbreaks have highlighted that even limited exposure can result in secondary transmission if biosafety protocols are not maintained (26, 27). Such exposures, especially in laboratory environments, can lead to serious outbreaks if biosafety protocols are not strictly followed. For this reason, culture handling and diagnostic procedures involving *Brucella* should be performed under Biosafety Level 3 (BSL-3) conditions, with the use of biological safety cabinets and appropriate personal protective equipment to minimize occupational hazards (28, 29).

Brucellosis pathogenesis involves complex interactions between bacteria and the host immune system (30). *Brucella* species are highly adaptable to evade immune responses, facilitating persistent infections (30). Once inside the host, *Brucella* survives and proliferates within macrophages, enabling widespread dissemination (31, 32). Its ability to manipulate host processes such as autophagy and apoptosis is central to persistence and replication (32, 33), making the intracellular environment a significant barrier to effective vaccine and therapeutic development.

Brucellosis diagnosis has traditionally relied on serological tests and culture methods; however, these methods can be limited by atypical clinical presentations and irregular bacterial distributions (34, 35). Advances in molecular diagnostics, such as polymerase chain reaction (PCR) and whole-genome sequencing (WGS), offer more rapid and accurate detection, thereby facilitating earlier treatment and improving patient outcomes (36, 37).

Although several therapeutic options exist, the emergence of antibiotic-resistant *Brucella* strains has become a growing concern (38, 39). Reducing disease transmission by eliminating potential animal carriers, especially cattle, may help control disease spread (40). Current treatment regimens typically involve multiple antibiotics, but treatment failure and relapse are still common, highlighting the need for novel therapeutic strategies (41). For optimal clinical outcomes, careful selection of effective antibacterial agents and appropriate treatment protocols is essential (42).

Despite advances in diagnosis and management, brucellosis continues to present substantial public health challenges. This review aims to explore disease pathogenesis, diagnostic methods, and therapeutic approaches, with a particular focus on transmission routes. Strengthened collaboration among public health authorities, clinicians, and veterinary professionals is essential to enhance prevention and control strategies and better understand the global impact of brucellosis.

## 2 Pathogenesis and adhesins of *Brucella* spp.

*Brucella* spp. can overcome various host defense mechanisms during the early stages of infection, during which the bacterial survival rate is approximately 10% (43). These pathogens have evolved sophisticated strategies to evade immune responses and can infect a range of cell types, including phagocytic cells such as macrophages and dendritic cells, as well as non-phagocytic cells such as epithelial cells and placental trophoblasts. Red and white blood cells (RBCs and WBCs), although not sites of replication, contribute to bacterial dissemination (31). A hallmark of *Brucella* pathogenicity is its ability to survive and replicate within macrophages, leading to chronic infections (44).

In animals, particularly cattle, sheep, and goats, *Brucella* infection is strongly associated with reproductive disorders such as abortion, retained placenta, orchitis, and infertility, which represent major veterinary and economic concerns (22, 45, 46). By contrast, in humans, spontaneous abortion is relatively uncommon; instead, brucellosis more frequently results in systemic and focal complications such as osteoarticular disease, endocarditis, and neurobrucellosis (47–50). This contrast underscores the divergent pathogenic outcomes and host-pathogen interactions between animal and human infections.

**Figure 1:**
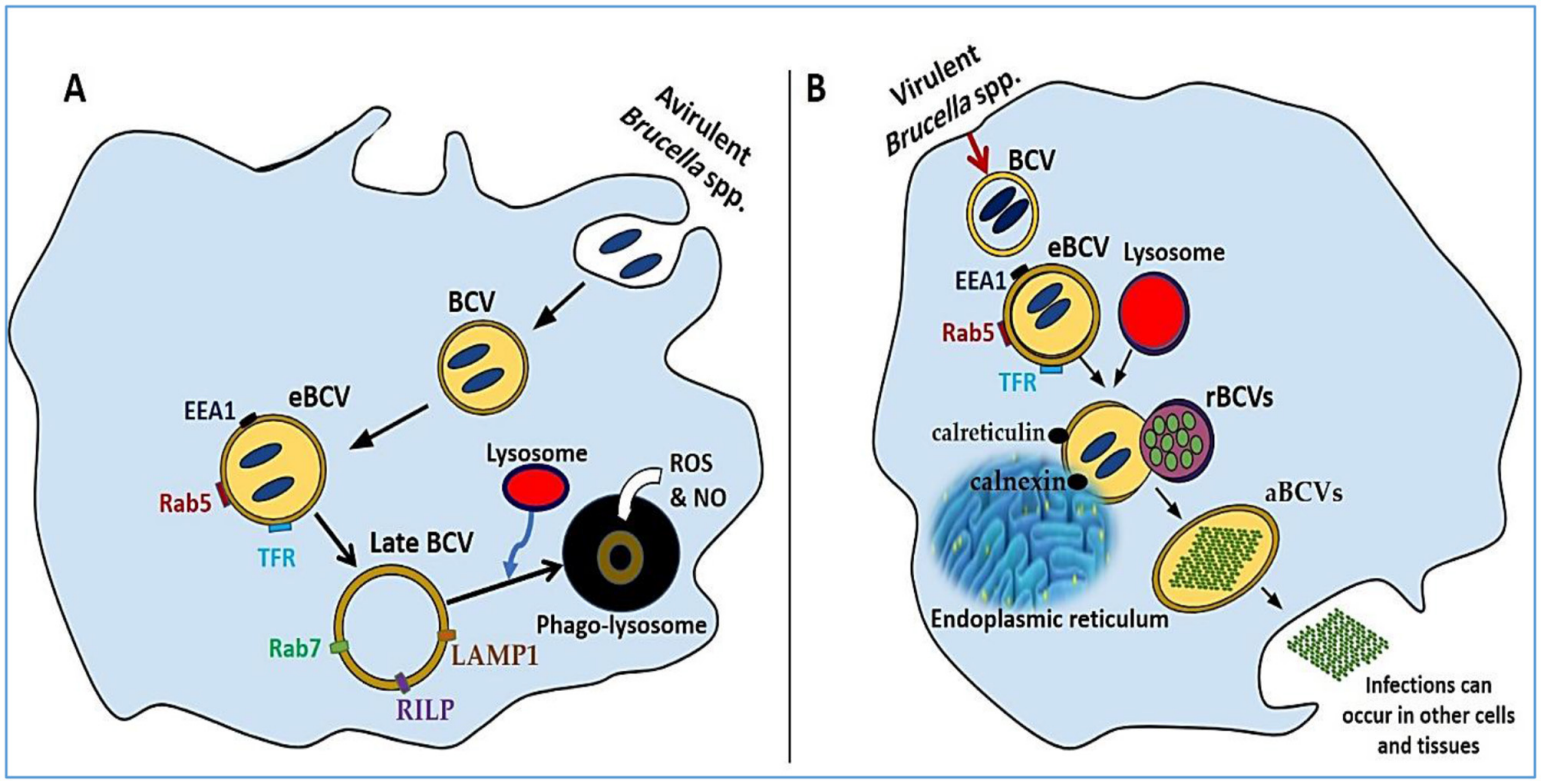
A comparative analysis of phagocytosis and exocytosis. **(A)** The intracellular development of an avirulent *Brucella* strain, highlighting the formation of eBCVs and late BCVs that eventually merge with lysosomes for bacterial degradation. **(B)** The progression of a virulent *Brucella* strain, showing evasion of lysosomal fusion and replication within the ER, followed by host cell lysis and dissemination to other tissues.

The internalization of *Brucella* into macrophages involves a zipper-like mechanism. Virulent strains preferentially enter through lipid rafts, whereas avirulent strains undergo phagocytosis, resulting in lysosomal fusion and degradation. This finding underscores the importance of lipid raft-mediated entry for intracellular survival during early infection (51, 52). Once inside the host cell, *Brucella* resides in membrane-bound vesicles known as *Brucella*-containing vacuoles (BCVs) (9, 43). These phagosomes evolve through stages, initially fusing with early endosomes to form early BCVs (eBCVs), which express markers such as early endosome antigen 1 (EEA1), Rab5, and transferrin receptor (TfR). Subsequently, fusion with late endosomes produces late BCVs containing lysosomal-associated membrane protein 1 (LAMP1), Rab7, and Rab-interacting lysosomal protein (RILP) (Figure 1A) (53).

In avirulent strains, these BCVs typically merge with lysosomes, where they are exposed to reactive oxygen species (ROS), nitric oxide (NO), and lysosomal antimicrobial peptides, ultimately leading to bacterial degradation (9, 54, 55). Conversely, smooth LPS *Brucella* strains evade lysosomal fusion. They achieve this by secreting muramidase and expressing SegA, a protein that blocks the maturation of eBCVs into degradative compartments (56). The type IV secretion system (T4SS) is also key to avoiding immune detection and enabling intracellular survival. Replicative BCVs (rBCVs) emerge through fusion with the endoplasmic reticulum (ER), where they acquire ER markers such as calnexin and calreticulin, where *Brucella* replicates and evades immune responses (Figure 1B) (52). Further adaptation leads to the formation of autophagic BCVs (aBCVs), which are marked by the expression of autophagy-related proteins such as ULK1, Beclin 1, and ATG14L, allowing long-term intracellular persistence (57). Once macrophages fail to control infection, they undergo lysis, releasing *Brucella* into adjacent tissues and facilitating systemic spread (58).

**Figure 2:**
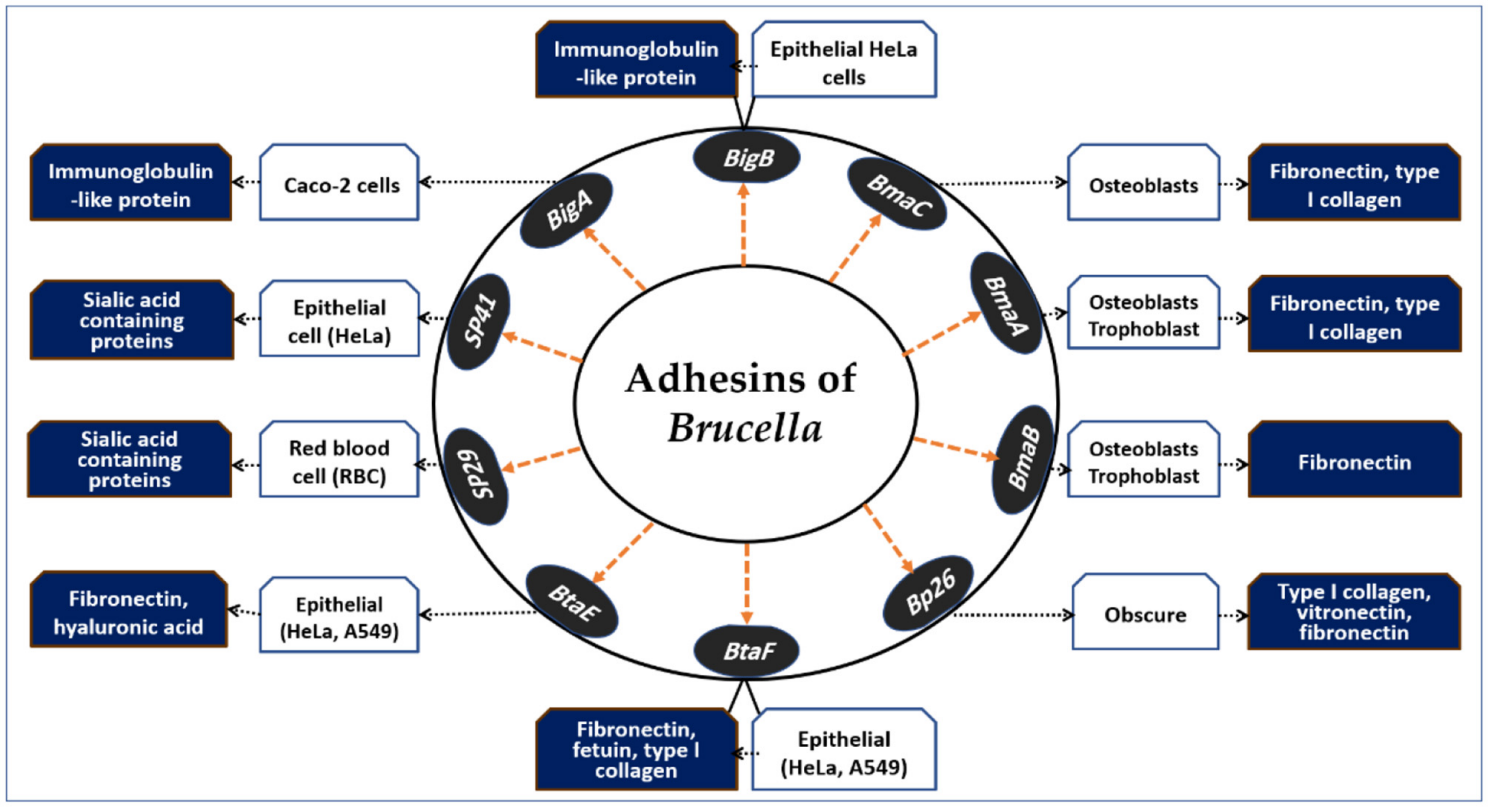
Adhesins of *Brucella* spp. and their host cell interactions. This schematic illustrates the major *Brucella* adhesins (*SP29, SP41, BigA, BigB, BmaA, BmaB, BmaC, Bp26, BtaE*, and *BtaF*) and their interactions with host cell types and receptors. Adhesins facilitate binding to epithelial cells, red blood cells, osteoblasts, and placental trophoblasts via host molecules such as sialic acid–containing proteins, fibronectin, vitronectin, hyaluronic acid, fetuin, and type I collagen. These adhesion mechanisms contribute to tissue tropism, colonization, and the intracellular persistence of *Brucella*, representing key steps in pathogenesis.

Brucellar adhesins play pivotal roles in host cell invasion. Although *Brucella* spp. lack fimbrial adhesin loci and do not form pilus-like structures under electron microscopy, several non-fimbrial adhesins that mediate adherence to host cells have been identified (59). A graphical overview of these adhesins and their receptor interactions is presented in Figure 2. The diagram highlights how distinct adhesins mediate attachment to a variety of host cell types, including epithelial cells, erythrocytes, osteoblasts, and placental trophoblasts. By exploiting host receptors such as sialic acid-containing proteins, fibronectin, vitronectin, hyaluronic acid, and type I collagen, *Brucella* ensures successful adhesion and invasion, which are critical for intracellular survival and dissemination.

Hemagglutination assays using RBCs have identified lectin-like adhesins. Rocha-Gracia et al. (60) reported that *B. abortus* and *B. melitensis* agglutinate erythrocytes from various species via a 29 kDa surface protein (*SP29*). Neuraminidase treatment reduced *SP29* binding to rabbit RBCs, suggesting that it interacts with sialic acid receptors. In *B. melitensis, SP29* likely functions as a D-ribose-binding periplasmic protein precursor. While this species can infect erythrocytes in murine models (61), further work is needed to define the *in vivo* role of *SP29*.

*SP41* was the first *Brucella* adhesin characterized *in vitro* (62). Antibodies against *SP41* reduced *B. suis* adhesion to HeLa cells, and deletion of the *ugpB* gene—which is implicated in *SP41* function—also diminished adhesion. Binding was inhibited by neuraminidase, highlighting a role for sialic acid residues. However, in *B. ovis, ugpB* deletion has no effect on adhesion or survival in macrophages or HeLa cells (63). Notably, the *ugpB* gene is functional in *B. ovis* but differs slightly from its homolog in *B. suis*. The lack of O-polysaccharide chains in *B. ovis* may indicate that alternative adhesins predominate.

The *bigA* gene, located on chromosome 1 of *B. abortus* (54), facilitates adherence to MDCK and Caco-2 cells. Czibener et al. (64) reported that this outer membrane adhesin, which contains an immunoglobulin-like domain, is essential for adherence. Deleting the pathogenic island BAB1_2009–2012 reduced adherence to HeLa cells. The *BAB1_2009* gene encodes *BigA*, which has a BIg-like domain found in invasin/intimin family adhesins (65). Preincubating bacteria with antibodies against this domain significantly decreased the number of intracellular bacteria in HeLa cells (64). Overexpression of *BigA* enhanced adhesion and invasion in polarized epithelial cell lines by promoting contact with cell–cell junctions and inducing cytoskeletal rearrangements. The same locus also expresses *BigB* (*BAB1_2012*) (66), and the ΔbigB mutation significantly reduced the number of intracellular bacteria in HeLa and polarized MDCK cells during early infection stages. Recombinant *BigB*, like *BigA*, alters the cytoskeleton and affects focal adhesion locations. The *BAB1_2011* gene encodes *PalA*, which is necessary for *BigA* and *BigB* expression, highlighting the role of the genomic island in *Brucella* adherence.

One study investigated *Brucella Bp26* as an in vitro adhesin and reported that it elicits significant antibody responses in infected individuals (67). *Bp26*, which is approximately 250 amino acids long and contains a poorly understood motif (DUF541), interacts with type I collagen, soluble vitronectin, and soluble fibronectin but not with laminin. Its role in *Brucella* cell attachment and *in vivo* infection effects remains unclear. *B. suis* 1330 contains the monomeric autotransporter proteins *BmaA* and *BmaB*, encoded by BR0173 and BR2013, which are smaller than *BmaC*. Compared with wild-type strains, mutants lacking *BmaB* are removed more quickly from the spleen of BALB/c mice, suggesting that *BmaB* plays a role in chronic infection (68). A recent study by Bialer et al. (69) indicated that the *bmaB* locus in *B. abortus* and the *bmaA* and *bmaC* loci in *B. melitensis* may be pseudogenes, although some reports suggest that Bma proteins may have functional roles in certain *B. suis* strains. *BmaA, BmaB*, and *BmaC* likely contribute to bacterial attachment to various cell types, indicating diversity in *Brucella* spp. adhesins and potential host preferences. The discovery of *BmaB* also suggests its involvement in cell division, generating a new pole (69).

The autotransporter adhesin (AT) is essential for bacterial adhesion to mammalian cells (70). *Brucella* possesses five AT adhesins: type I monomeric ATs *OmaA* and *BmaC* (71), type II trimeric ATs *BtaE* and *BtaF* (72, 73), and the inverted AT adhesin *BigA* (64). *BmaC* specifically binds fibronectin (71), whereas *BtaE* and *BtaF* bind hyaluronic acid (72, 74). Mutants lacking these adhesins exhibit reduced adhesion to epithelial cells but maintain wild-type macrophage replication. In mice, these mutations are reversed when AT adhesins are administered intragastrically or nasally, indicating their role in mucosal adhesion. Some pathogens, such as *BigA*, exploit eukaryotic cell junctions to breach mucosal barriers (75). A double mutant of *B. suis btaE btaF* is more attenuated than a single mutant, suggesting complementary virulence roles (76). *BtaF* also shields *B. suis* from serum bactericidal action (72). *BmaC, BtaE*, and *BtaF* are localized near the cell pole (71, 74) and form a binding pole with G1 phase *Brucella* cells (77). In planktonic cultures, *Brucella* produces these adhesins in limited amounts, resulting in effective gene transcription during interactions with human cells. Several AT-encoding genes are regulated by *VjbR* (78) and *MucR* (79, 80), whereas *btaE* expression in *B. abortus* is controlled by a complex regulatory network (81, 82). *Brucella* AT-type adhesins may have multiple functions (82), necessitating cross-species and strain studies using mutants with gene disruptions to clarify their role in pathogenicity.

## 3 Diagnosis of brucellosis

Timely and accurate diagnosis is critical for the effective treatment and control of brucellosis. Current diagnostic tools include a range of serological, culture-based, and molecular methods. Serological assays, such as the Rose Bengal test and ELISA, offer rapid screening capabilities but can be limited in both sensitivity and specificity. Blood culture remains the gold standard for definitive diagnosis; however, it is time-consuming and may yield false negatives, particularly in patients who have already begun antibiotic therapy. Molecular approaches, including PCR and WGS, offer faster detection of *Brucella* DNA and are particularly valuable when traditional methods fall short. These complementary techniques collectively increase diagnostic accuracy, guide treatment decisions and improve patient outcomes. The following section outlines both current and emerging diagnostic approaches for brucellosis in humans and animals.

Recent advances in proteomics are reshaping brucellosis research and its applications in diagnosis, prevention, and control. In clinical microbiology, MALDI-TOF MS has emerged as a powerful tool for rapid, species-level discrimination of *Brucella* (e.g., *B. abortus* vs. *B. melitensis*) based on whole-cell proteomic fingerprints. The continuous expansion of spectral databases for highly pathogenic bacteria is closing gaps that previously limited diagnostic coverage, while machine-learning approaches applied to spectral data are further improving the classification of closely related species (83–85). Large-scale LC–MS/MS proteomic analyses are also generating serum biomarker panels capable of distinguishing acute from chronic brucellosis, with network-based and machine-learning methods offering promising candidates for future clinical assays (86, 87). In addition, immuno-proteomics has identified type IV secretion system components and outer-membrane proteins with high diagnostic sensitivity and specificity, and the design of multi-epitope fusion proteins from proteome-mined antigens is advancing serological testing while reducing the problem of LPS cross-reactivity (88, 89).

On the prevention and control side, proteomic prioritization of conserved outer-membrane proteins (e.g., *Omp16, Omp25/BP26*) supports the development of next-generation vaccines, including mRNA and outer-membrane vesicle (OMV)-based platforms, with OMV proteomes revealing multiple protective antigens (90, 91). Finally, pan-proteomic studies of reference and field isolates using label-free quantitation are identifying conserved stress-responsive proteins as potential biomarkers for surveillance and intervention, while innovations such as magnetics-assisted MALDI workflows point toward future culture-independent detection strategies for high-risk pathogens including *Brucella* (92, 93). These advances in proteomics complement conventional diagnostic modalities and highlight the ongoing evolution of brucellosis diagnostics; the following subsections detail the established culture-, serology-, and molecular-based methods that remain central to routine practice.

### 3.1 Culture methods

Accurate identification of *Brucella* species—the causative agents of zoonotic brucellosis—relies on isolation of the pathogen from blood, bone marrow, or other tissues (37). The success of culture-based detection varies according to disease stage, sample type, prior antimicrobial exposure, and culture technique used (94). Despite its limited sensitivity, culture remains the most definitive method of diagnosis (95). Innovations such as advanced incubators and the Ruiz-Castañeda biphasic culture system have improved biosafety and fostered more reliable bacterial growth. When performed promptly upon clinical suspicion, peripheral blood cultures are crucial for confirming the diagnosis, with reported sensitivities ranging from 10% to 90% (14, 96). These cultures are especially valuable when serological results are inconclusive (97). The techniques used include manual culture, lysis-based systems (98), clot cultures, and automated platforms—each contributing to increased sensitivity and faster detection (99).

During early infection, the bacterial load in the bloodstream is typically low and may be missed if the sample size is insufficient. To maximize diagnostic yield, it is recommended that two or three separate peripheral blood cultures be obtained (100). As brucellosis progresses, the bacterial burden often decreases, complicating pathogen isolation (101). Given the slow growth rate of *Brucella*, culture protocols must be extended to accommodate delayed detection (102). In severe cases, traditional culture methods may require incubation for up to 7 days, whereas automated systems may detect growth within 5 days (103). The American Society for Microbiology and the World Health Organization advocate for a 1-month incubation period for blood culture bottles, although this recommendation can pose logistical and financial challenges (104).

Within 24 h of infection, an estimated 25%−35% of patients may exhibit dissemination of *Brucella* beyond the bloodstream. Cultures may also be performed from bone marrow, urine, liver biopsies, lymph nodes, and cerebrospinal fluid and incubated at 35 °C in 5% CO_2_ for up to 2 weeks (105). Confirming the identity of *Brucella* species is essential for mitigating biosafety risks. Classic identification methods include phage lysis testing, oxidative metabolism assays, and agglutination with monospecific antisera (37). Owing to the limitations of conventional culture, serological testing can be employed to increase sensitivity. In recent years, matrix-assisted laser desorption ionization time-of-flight mass spectrometry (MALDI-TOF MS) has emerged as a rapid, non-phenotypic identification method (106, 107). However, prior genomic validation is needed to ensure accuracy (39). Many laboratories now apply MALDI-TOF MS by directly introducing broth from positive cultures into the matrix (108, 109). *Brucella* organisms are safely inactivated via 100% ethanol prior to protein extraction, minimizing the risk of laboratory exposure (110).

MALDI-TOF MS has been used to identify *Brucella* reference strains from synthetic blood cultures (111). A refined Vitek MS database—including 590 protein spectra from 84 *Brucella* isolates—facilitates discrimination between *Brucella* and *Ochrobactrum* species, as well as accurate identification of *B. abortus, B. melitensis*, and *B. suis*. Further validation using wild-type isolates from diverse geographic and host sources is necessary. While the cost per sample with MALDI-TOF MS is relatively low, the initial investment and operational expenses can restrict access in endemic regions with limited resources (110).

*Brucella* species are among the most common causes of laboratory-acquired infections and are capable of causing outbreaks if proper containment measures are not enforced (112). Laboratory workers face significant risk due to the aerosolized particles generated during specimen handling. The routes of infection include inhalation, mucosal exposure, ingestion, and percutaneous entry. Reported infection rates among clinical laboratory staff range from 10% to 100%, influenced by pathogen load and laboratory safety standards (113, 114). Early-phase blood cultures, if misinterpreted by Gram staining, can lead to diagnostic errors due to *Brucella*'s subtle morphology (115).

Inadequate biosafety protocols, particularly in resource-limited settings, increase the risk of laboratory-acquired infections (116). For example, one Turkish laboratory reported an 18% infection rate among staff, with an annual risk of 8% (116). Effective communication between clinicians and microbiologists is essential to ensure proper identification and handling of suspected *Brucella* samples. Until a diagnosis is confirmed or ruled out, all potentially hazardous samples should be managed with heightened containment and stored appropriately to prevent accidental exposure (117).

### 3.2 Serological methods

The primary diagnostic tools for brucellosis include culture, serological assays, and molecular techniques (3, 104). Given the nonspecific clinical presentation of brucellosis, laboratory confirmation is essential (118). Although serological methods are widely employed for identifying *Brucella* infections, their accuracy can be affected by limited sensitivity, cross-reactivity with other pathogens, and the need for well-equipped laboratories (3). In low-resource settings or in areas with lower disease prevalence, serological testing remains the cornerstone of diagnosis because of its relative simplicity, affordability, and high negative predictive value (104). Nonetheless, interpreting serological results can be challenging and sometimes inconclusive (118).

Common serological assays for diagnosing human brucellosis include the serum agglutination test (SAT), Rose Bengal test (RBT), Coombs test, and enzyme-linked immunosorbent assay (ELISA), which are generally ranked in the following order: ELISA > RBT > SAT > Coombs test (119). Compared with SAT or RBT, ELISA offers greater sensitivity (120–122). The performance of ELISA depends on the specific immunoglobulin detected. For example, Araj et al. (122) reported 91% sensitivity for IgG and 100% sensitivity for IgM, both with 100% specificity. In contrast, Memish et al. (123) reported lower IgG sensitivity (45.5%) but similarly high specificity (97.1%); IgM showed 79% sensitivity and 100% specificity. Overall, the combined ELISA results had a sensitivity of 94.1% and a specificity of 97.1%. Xu et al. (124) reported a sensitivity of 88.37% for IgG and 74.42% for IgM, matching the sensitivity of SAT. When the IgG and IgM data were combined, the sensitivity increased to 98.84%, whereas the specificity decreased to 84.13% (121, 125). These results suggest that while ELISA has excellent sensitivity (119), its reduced specificity may limit its standalone diagnostic utility (126).

As the disease progresses, IgG antibodies may become non-agglutinating (127). The Coombs test helps detect blocking antibodies in such cases, although it is infrequently used owing to its technical demands and the requirement for trained personnel. Alternatively, the Brucellacapt test detects both agglutinating and non-agglutinating antibodies (120) and may serve as a practical replacement for the Coombs test (120, 128). Xu et al. (124) demonstrated the increased specificity of Brucellacapt for diagnosing human brucellosis. Ardic et al. (129) reported a sensitivity of 97.3%, specificity of 55.6%, positive predictive value of 90%, and negative predictive value of 83.3% at a 1:160 titer. The test performance varied depending on the disease stage. A titer of 1:160 was considered optimal by Xu et al. (124), while increasing the threshold to 1:320 reduced the sensitivity. Although Brucellacapt can help detect chronic brucellosis (120), it may yield negative results in some chronic cases (124). An effective serological diagnostic strategy requires a highly sensitive test followed by a confirmatory assay (130). Xu et al. (124) reported that ELISA, with a sensitivity of 98.84% and a negative predictive value of 98.15%, is effective for rapid screening, especially in endemic regions. Brucellacapt offers excellent specificity and positive predictive value, making the combination of ELISA and Brucellacapt highly beneficial for diagnosing brucellosis in resource-limited and high-burden settings.

The RBT is a rapid, card-based agglutination assay that detects both agglutinating and non-agglutinating antibodies, yielding qualitative results (104). Performing RBT with serum dilutions can improve the specificity for samples initially testing positive (131, 132). However, false-positive results may occur due to factors such as hemolyzed serum, prior exposure, non-specific antibody binding, or cross-reactivity. This is particularly problematic in low-endemic areas, where the reduced positive predictive value of serological testing may lead to unnecessary follow-up, increased healthcare costs, and patient anxiety (133). Test accuracy can also be influenced by the disease stage, immune status of the host, and specific *Brucella* species involved. Laboratory-related errors further highlight the need for proper training and stringent quality assurance practices (104).

Because of the complexity of *Brucella* antigenic structures, various immunological approaches are employed for diagnosis (37). Whole-cell antigens are used in indirect fluorescent antibody tests (134). Most serological assays target antibodies against smooth LPS or cytosolic proteins. The immune response to smooth LPS—common to smooth *Brucella* species—results in sequential production of IgM (first week) and IgG1 (second week), followed by IgG2 and IgA (third week) (104). Misdiagnosis may occur with species such as *B. canis*, which lack O-polysaccharides, complicating detection in human infections (135). To address these diagnostic limitations, Loubet et al. (133) conducted a retrospective study at the French National Reference Center for *Brucella*, analyzing 3,587 serum samples from June 2012 to June 2023. Among these cases, 148 were confirmed brucellosis cases. Although individual tests exhibited high sensitivity and specificity, the diagnostic accuracy improved significantly when the assays were combined. The best-performing algorithm—using RBT, Brucellacapt, and ELISA for IgM and IgG—achieved a sensitivity of 90.5% and specificity of 99.7%. These findings underscore the importance of integrated diagnostic strategies and the need for continued innovation in testing methods.

In animals, brucellosis is primarily diagnosed via serological assays such as RBT, complement fixation tests (CFTs), and ELISA (136). Although these tests effectively detect *Brucella*-specific antibodies, their reliability diminishes in chronic infections, when antibody titers often fall below detectable levels (137). Furthermore, *Brucella*'s ability to survive intracellularly enables the pathogen to evade immune detection, complicating serological diagnosis (138). As a result, seronegative carriers—infected animals that do not produce detectable antibodies—pose a serious challenge for disease control, as they can still transmit the infection to other animals and humans (139). To address these diagnostic gaps, molecular techniques such as PCR should be used alongside serological methods to detect and manage brucellosis accurately in both humans and animals.

### 3.3 Molecular methods

Molecular diagnostic technologies, particularly PCR, have gained significant prominence in the detection and identification of *Brucella* species (138). PCR offers high sensitivity and specificity, enabling the detection of Brucella DNA in various biological samples, including blood, milk, tissues, and semen (136). Unlike serological tests, which detect host antibody responses, PCR directly targets *Brucella* DNA, making it particularly valuable for identifying infections in seronegative individuals and animals. Among the molecular targets, the insertion sequence IS711 is widely utilized because of its specificity for the *Brucella* genus (140). PCR assays based on this gene have demonstrated high diagnostic utility, especially in cases where culture fails or serological tests yield negative results. For example, Hinić et al. (141) demonstrated that IS711-based PCR could detect *Brucella* DNA in wild boars even when traditional isolation methods were unsuccessful and serological tests were negative. The ability of PCR to amplify DNA from a variety of sample types underscores its importance in endemic regions where rapid and accurate diagnosis is critical. This is particularly relevant in scenarios where serological tests are limited by low sensitivity or delayed antibody responses (133).

Several outer membrane protein (OMP) genes, including *omp2, omp31*, and *omp28* (Bp26), which serve as additional targets for PCR-based detection, have also been identified through molecular diagnostics (142, 143). Although 16S rRNA and IS711 remain widely used for *Brucella* identification, some studies have raised concerns regarding IS711′s variability and occasional deletions in certain strains, which may affect assay sensitivity. Another widely used marker is the *bcsp31* gene, which encodes a highly immunogenic membrane protein and has been validated for reliable species identification (142, 144, 145). Multiple PCR-based techniques, including conventional PCR, real-time PCR, multiplex PCR, nested PCR, and PCR-enzyme immunoassays in microplate formats, have been developed to increase diagnostic performance (104). Multiplex PCR is particularly advantageous, as it allows for simultaneous detection and differentiation of field strains and vaccine strains such as S19, RB51, and Rev.1 in a single assay (146, 147).

In recent years, loop-mediated isothermal amplification (LAMP) has emerged as a promising alternative to PCR. LAMP offers several advantages, including rapid amplification, visual detection of results, and minimal equipment requirements—typically only a constant-temperature heat source such as a 63 °C water bath. This method eliminates the need for gel electrophoresis and produces results in under 1 h, making it highly suitable for field diagnostics and use in low-resource settings (148). Its affordability and ease of use make it an attractive option for point-of-care testing in brucellosis-endemic regions.

Sequencing-based technologies also contribute to an improved understanding of *Brucella* epidemiology. Whole-genome sequencing and other genetic analyses have elucidated the mechanisms underlying strain variation, virulence, and evolutionary relationships (149, 150). Such data are critical for advancing vaccine development and refining diagnostic targets (151). However, the high cost and technological demands of next-generation sequencing limit its widespread application, especially in low-income countries where brucellosis is often endemic (150, 152, 153).

Emerging molecular innovations aim to overcome these barriers. Magnetic nanoparticle-based DNA biosensors have shown potential for rapid and highly specific detection of *Brucella* DNA. These biosensors employ frequency-mixing magnetic detection and DNA hybridization, enabling the identification of low DNA concentrations within minutes—even in field conditions (138, 154). Additionally, immuno-surface plasmon resonance biosensors have been developed to detect *Brucella* without the need for DNA amplification. These portable, cost-effective devices offer a detection threshold as low as 2.8 bacteria/ml, presenting a promising solution for decentralized testing (155).

Despite the increasing availability of molecular tools, traditional culture and serological methods remain the standard diagnostic approaches for brucellosis in many settings. However, the diagnosis remains challenging because of the disease's non-specific symptoms, which often resemble those of other febrile illnesses and can result in delayed or missed diagnoses (156). The lack of pathognomonic clinical signs, combined with the risk of false-negative serological results in early or atypical presentations, highlights the need for improved diagnostic awareness (95, 157). Education of clinicians and health workers, especially in endemic areas and among at-risk populations, is critical for enhancing early recognition and response to brucellosis (158).

## 4 Pathways of brucellosis transmission

**Figure 3:**
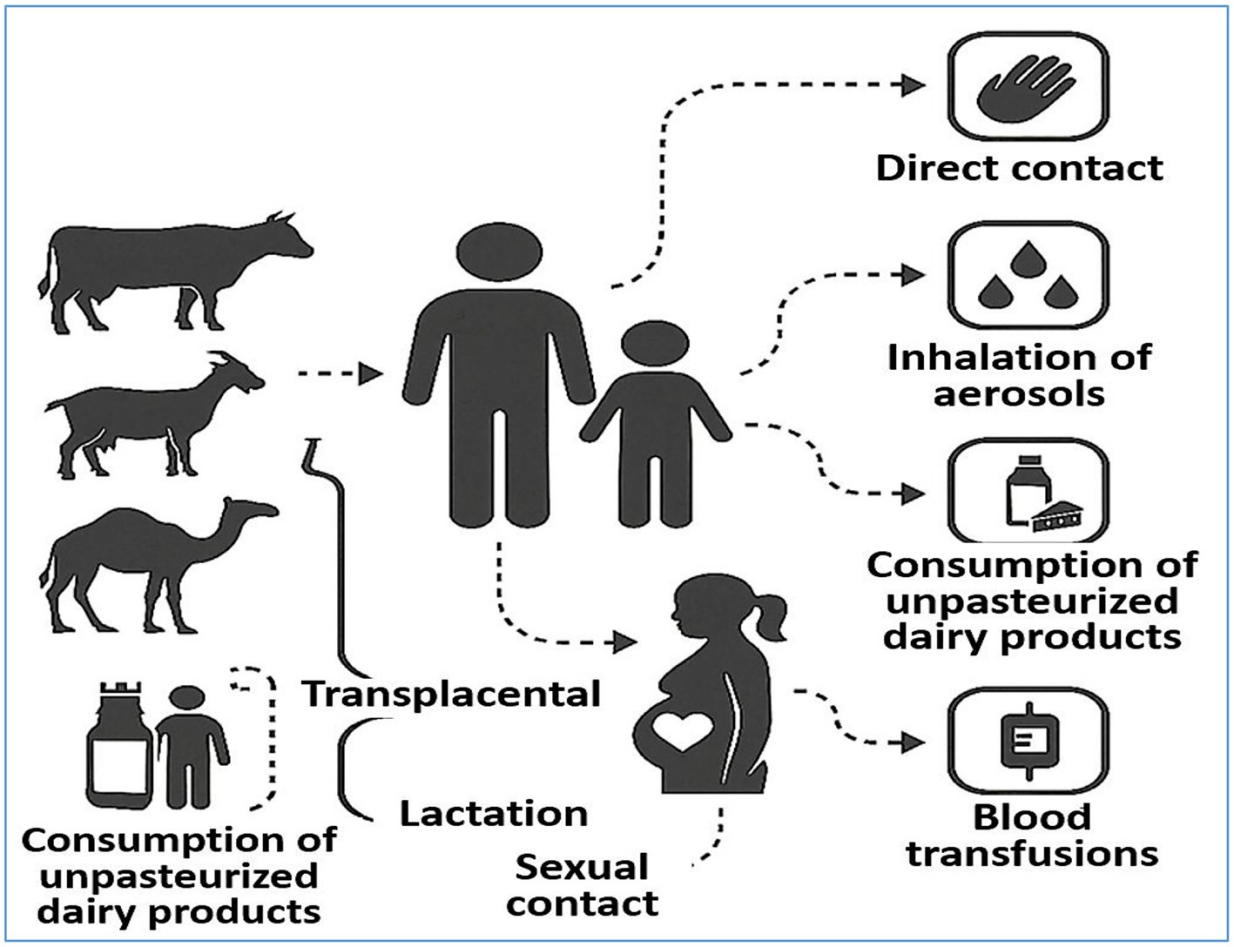
Transmission Pathways of Brucellosis. The diagram illustrates the major transmission routes of *Brucella* spp. from animals to humans, including direct contact with infected animals, ingestion of unpasteurized dairy products, inhalation of contaminated aerosols, and exposure through mucous membranes or broken skin. It also highlights less common human-to-human transmission pathways such as transplacental transfer, breastfeeding, sexual contact, blood transfusion, and organ transplantation. Understanding these routes is critical for developing effective prevention and control strategies.

In addition to implementing accurate diagnostic strategies, identifying and disrupting the transmission pathways of brucellosis to effectively control and prevent the disease is imperative (159). Brucellosis is primarily a zoonotic disease transmitted from animals to humans, although human-to-human transmission—while rare—has also been documented (160). Although the latter route is less common, both pathways contribute to the persistence and potential expansion of brucellosis, emphasizing the need for comprehensive preventive measures (Figure 3).

Zoonotic transmission typically occurs through direct contact with infected animals or their secretions, especially during the handling of aborted fetuses, placental tissues, or birth fluids. Occupational exposure is a significant risk factor, particularly for farmers, veterinarians, abattoir workers, and laboratory personnel. Inhalation of infectious aerosols—especially in confined environments such as laboratories and livestock facilities—is another important mode of transmission (161, 162). Moreover, the ingestion of unpasteurized milk, cheese, and other dairy products derived from infected animals remains a major source of human brucellosis, particularly in endemic regions where food safety regulations are inadequately enforced (162).

Although infrequent, human-to-human transmission via several mechanisms has been reported. These include vertical transmission across the placenta, breastfeeding, sexual contact, and iatrogenic exposure through contaminated blood transfusions or bone marrow transplantation (160). Aerosol transmission has also been implicated in clinical and laboratory settings under specific conditions (96). Although such cases are uncommon, the wide array of possible transmission routes expands the pool of susceptible individuals and necessitates vigilance across multiple sectors of public health and clinical care.

A diverse range of animals serve as reservoirs for *Brucella* species, including cattle (*B. abortus*), goats and sheep (*B. melitensis*), swine (*B. suis*), camels, dogs (*B. canis*), poultry, and numerous wildlife species (163). These hosts play crucial roles in maintaining the endemicity of brucellosis and facilitating its transmission to humans. Human infection is not restricted by age or sex; however, young and middle-aged adults are most frequently affected by increased occupational and environmental exposure (164). Pregnant women and newborns also remain vulnerable, given the potential for transplacental transmission and perinatal complications (165, 166).

## 5 Brucellosis treatment regimens

Brucellosis treatment has evolved significantly since the mid-19th century. Early therapeutic attempts—dating back to 1855—included quinine, colchicine, and ampicillin, followed by the use of salicylates, ichthyol, iodine, immune sera, and early vaccines. However, these treatments often lack efficacy and are associated with considerable toxicity (162). Sulfonamide drugs were introduced in 1936, marking the beginning of antimicrobial therapy for brucellosis, although the results have been inconsistent (9). The addition of streptomycin in the late 1940s, used alone or combined with oral sulfadiazine, also failed to achieve consistently successful outcomes (161).

Subsequent studies demonstrated that combination antibiotic therapy produced significantly better results than monotherapy, reducing relapse rates and improving overall efficacy (161). In 1971, the World Health Organization (WHO) recommended a 3-week treatment course comprising tetracycline and streptomycin. This protocol was revised in 1986 to recommend a 6-week regimen of doxycycline and rifampicin or a 2 to 3-week course of tetracycline plus streptomycin, which has become the standard treatment approach for human brucellosis (167). Today, the cornerstone of brucellosis treatment remains antimicrobial therapy, particularly the use of dual antibiotics such as doxycycline (100 mg twice daily for 6 weeks) in combination with either streptomycin (1 g intramuscularly daily for 2–3 weeks) or rifampicin (600–900 mg daily for 6 weeks) (167, 168). The choice of regimen depends on the disease severity, patient comorbidities, and the presence of focal complications such as osteoarticular involvement or neurobrucellosis, which may require extended or adjusted courses of therapy.

**Figure 4:**
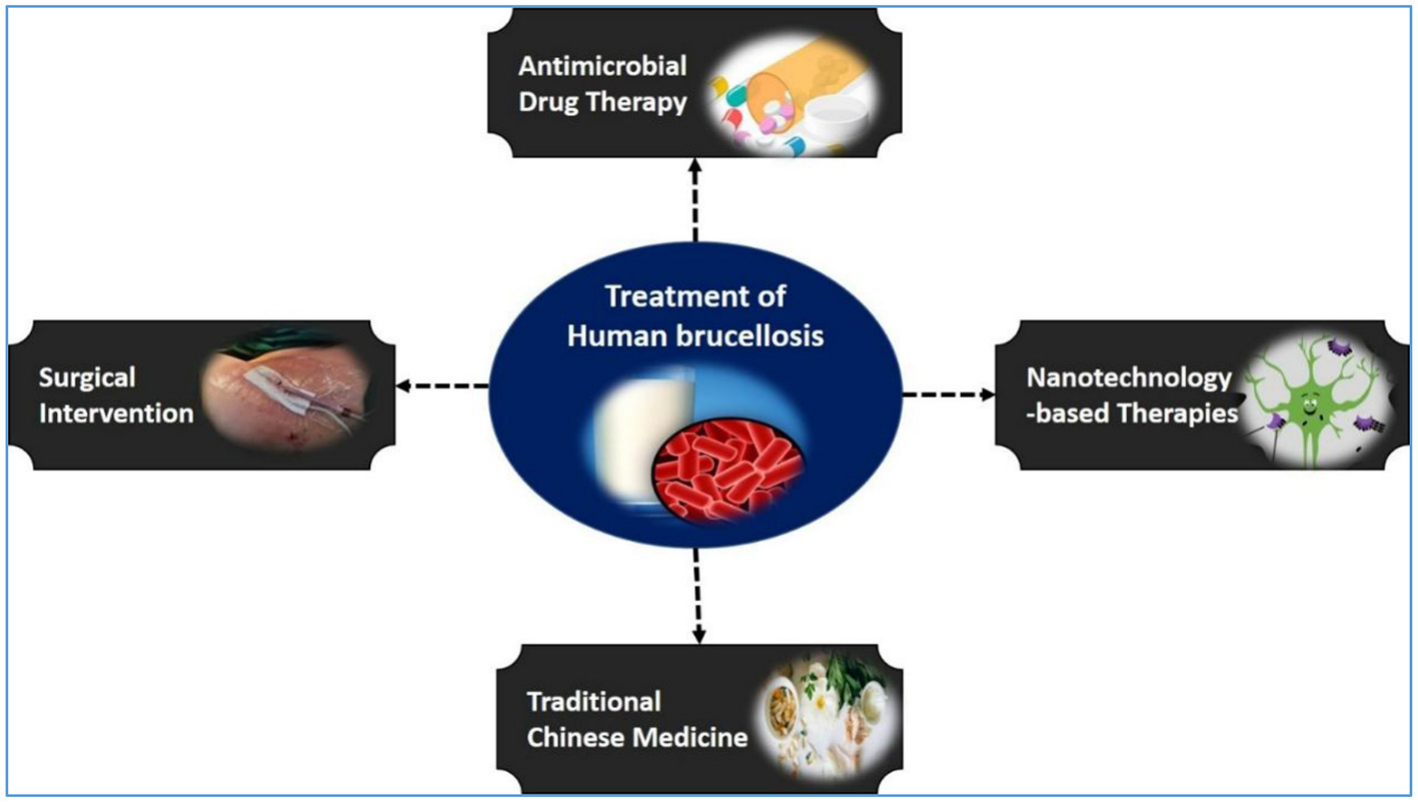
Treatment strategies for human brucellosis. A schematic overview showing the main therapeutic approaches for human brucellosis, including antimicrobial drug therapy, surgical intervention, traditional Chinese medicine, and emerging nanotechnology-based therapies.

Effective treatment is critical not only for resolving infection but also for minimizing the risks of chronic disease, relapse, complications, and transmission (Figure 4). Prompt therapy can reduce the incubation period, accelerate symptom relief, and lower both morbidity and mortality rates (169, 170). In addition to standard antibiotic regimens, adjunctive approaches—such as surgical intervention for severe cases, traditional Chinese and integrative medicines to enhance the immune response, and nanotechnology-based therapies for targeted drug delivery—are increasingly explored. These strategies underscore the importance of individualized treatment plans tailored to disease severity, comorbidities, and available resources. This section reviews each modality, outlining its key benefits and limitations.

### 5.1 Antimicrobial drug therapy

As no licensed vaccine exists for human brucellosis, antibiotic therapy remains the cornerstone of treatment (171). The intracellular nature of *Brucella*, particularly its residence within reticuloendothelial cells and bone, poses significant challenges for effective antibiotic penetration (172). Consequently, combination antimicrobial regimens that can penetrate macrophages and maintain efficacy in acidic environments are standard practices (173–175). Earlier monotherapies, including tetracycline, rifampin, and quinolones, had limited success, with high relapse rates; for example, ciprofloxacin alone was associated with an 83% recurrence rate (176). The WHO first recommended combination therapy in 1976, advocating for a 6-week course of rifampin and doxycycline (177), with other common regimens, including doxycycline plus streptomycin, rifampin, or trimethoprim-sulfamethoxazole (176). While 6-week treatments are generally recommended to reduce relapse (171), recent evidence suggests comparable outcomes with 4-week regimens using doxycycline, streptomycin, and hydroxychloroquine (178), potentially offering shorter, safer treatment options.

The current WHO and CDC guidelines endorse dual or triple antibiotic regimens (41). Triple therapy can reduce *Brucella* DNA levels more significantly (179). It is associated with higher rates of adverse effects, lower adherence, and increased risk of resistance (180). Injectable aminoglycosides such as gentamicin or streptomycin, often part of triple therapy, require parenteral administration and can limit feasibility in outpatient settings. Recent studies, including one conducted in Saudi Arabia, reported no significant differences in cure rates between dual and triple therapies (181). A 2025 study by Alsowaida et al. (41) further confirmed that dual therapy is equally effective but better tolerated, making it more cost-effective and preferable for most patients.

Despite these advances, several challenges remain. Routine antibiotic susceptibility testing is often bypassed due to safety risks to laboratory personnel and a lack of standardized testing protocols (182). Some antibiotics lack approval or defined breakpoints by the EUCAST or CLSI, complicating treatment decisions. High rifampicin resistance in regions with endemic tuberculosis, along with 5%−16% relapse rates, further complicates management (183–185). Severe cases involving osteoarticular infections, neurobrucellosis, or endocarditis require intensive therapy (186). Tetracyclines are contraindicated in young children and lactating women (187), and fluoroquinolones should not be used as monotherapy due to high relapse rates (188–190). While doxycycline remains the preferred agent, resource-limited settings may require alternative tetracyclines. Distinguishing relapse from reinfection remains a diagnostic challenge, emphasizing the need for timely and appropriate therapy (191). Antimicrobial therapy offers high cure rates and structured protocols but must be balanced against recurrence risk, side effects, and the emergence of resistance (175–178).

### 5.2 Surgical treatment

Surgical intervention is an important adjunct to antimicrobial therapy in cases where medical management alone is insufficient or when complications arise. In *Brucella* endocarditis, early antibiotic treatment combined with valve surgery significantly improves prognosis, reduces mortality, and enhances quality of life (192, 193). A study by Keshtkar-Jahromi et al. (194) involving 308 patients revealed that combining surgery with medical therapy lowered mortality from 32.7% to 6.7%. Surgery is indicated in cases of advanced heart failure, hemodynamic instability, prosthetic valve endocarditis, persistent bacteremia, valve dysfunction, local abscess formation, sinus tracts, and vegetation ≥30 mm—or >10 mm if highly mobile—despite adequate antimicrobial therapy (195, 196). For example, Hong et al. (197) reported a case where antibiotic therapy initially managed a small vegetation (<10 mm), but progression to mitral valve dysfunction required delayed surgical intervention. Postoperative antibiotic therapy was continued for 6 weeks, followed by lifelong prophylaxis.

In *Brucella* spondylodiscitis, long-term antibiotic therapy is the primary treatment, although surgery may be necessary in 3%−29% of cases (198, 199). Indications for surgical intervention include neurological deficits, large paravertebral or epidural abscesses unresponsive to medical therapy, spinal instability, or deformity (200, 201). While limited data exist on the surgical management of *Brucella* spondylitis, studies suggest that spinal instrumentation can be safely employed in infected patients without impeding bacterial eradication (201–203). Jiang et al. (204) suggested the combination of surgery with antibiotics such as rifampin and doxycycline. However, Katonis et al. (201). noted that chemotherapy alone is often effective and that surgery should be reserved for refractory or complicated cases. Surgical management is particularly beneficial for patients with extensive intervertebral disc damage, vertebral collapse, neurological deterioration, or spinal deformities. Postsurgical care necessitates extended antibiotic courses, typically exceeding 6 months, to prevent relapse and ensure full recovery.

### 5.3 Nanotechnology-based therapies

Despite the efficacy of conventional antimicrobial regimens, brucellosis frequently relapses owing to the ability of *Brucella* spp. to persist intracellularly within macrophages. This persistence impedes immune clearance and restricts antibiotic penetration (205). Nanotechnology offers a promising approach to overcome these limitations by enhancing drug delivery, reducing recurrence, and addressing antimicrobial resistance (206). NPs possess unique physicochemical properties that facilitate membrane penetration and enable targeted disruption of bacterial metabolic pathways (207, 208). NPs may function as intrinsic antimicrobials or act as delivery vehicles—referred to as nanobiotics or nanoantibiotics—for traditional antibiotics (207). Inorganic NPs with antimicrobial activity are termed nanobacteriocides, while those used to transport drugs are known as nanocarriers (207). These systems can bypass common resistance mechanisms, such as poor intracellular access and bacterial efflux pumps, which limits the effectiveness of standard antimicrobial agents (207, 209).

Several nanocarrier systems—such as solid lipid NPs, liposomes, chitosan-based NPs, niosomes, and their combinations with sodium alginate—have demonstrated potential for improving treatment outcomes in patients with brucellosis (210). For example, hydroxychloroquine and doxycycline delivered via solid lipid NPs combined with cadmium telluride quantum dots exhibited enhanced efficacy and may reduce relapse rates (211). In a study by Hosseini et al. (205), compared with free doxycycline, doxycycline-loaded solid lipid NPs reduced the intracellular burden of *B. melitensis* in macrophages by 3.5 logs, supporting their potential for preventing recurrence.

Codelivery strategies further improve outcomes. Curcumin, which has pH-sensitive antimicrobial activity, can potentiate doxycycline under acidic conditions (212). El-Essa et al. (213) assessed pH-responsive chitosan-sodium alginate NPs loaded with doxycycline and a curcumin-loaded niosome hydrogel in guinea pigs infected with *B. melitensis* biovar 3. This dual nanoformulation reduced the splenic bacterial load to 19 ± 3.0 log CFU, whereas it was 640.66 ± 4.3 log CFU in the untreated controls. Polyanhydride-based NPs encapsulating doxycycline and rifampicin have also shown promise. Lueth et al. (206) reported that these NPs, ranging from 162.8 to 326.8 nm in size, with polydispersity indices of 0.1–0.13 and zeta potentials of −1.56 to −21.2 mV, provided extended-release delivery. Over 5 days, they eradicated *B. melitensis* from infected macrophages and significantly reduced liver bacterial counts in BALB/c mice. Notably, no significant difference was observed between animals treated with daily free drugs and those treated weekly with nanoformulations, suggesting similar efficacy with a reduced dosing frequency.

The use of gentamicin, a potent but nephrotoxic antibiotic, can be enhanced via the use of nanocarriers (214, 215). Poly(lactic–coglycolic acid) (PLGA) microparticles and NPs (~1 μm and ~299 nm, respectively), which are coencapsulated with gentamicin and bis(2-ethylhexyl) sulfosuccinate sodium salt, reduce splenic infection by 3.23 logs and achieve 50% eradication in mice without renal toxicity (216). Poly(amidoamine) (PAMAM) dendrimers—water-soluble, hyperbranched polymers (1–15 nm, 30–200 kDa)—are another promising platform (217, 218). These nanocarriers (generations G0–G5) can deliver drugs or genetic material (219). Gentamicin-loaded G4 dendrimers modified with polyethylene glycol produced NPs with a diameter of 51.23 nm, a zeta potential of −8.8 mV, and a 0.2 polydispersity index. Enhanced intracellular drug release can be achieved via glutathione-mediated mechanisms and efflux pump inhibition (220).

Overall, nanoblass-based strategies—including microspheres, dendrimers, chitosan particles, and PEGylated formulations—offer targeted delivery, reduce toxicity, and improve efficacy for treating brucellosis. These advancements could revolutionize brucellosis therapy in both human and veterinary medicine by overcoming current limitations in antibiotic delivery and persistence (212).

### 5.4 Traditional Chinese medicine

Traditional Chinese medicine (TCM) has long been utilized for the treatment of infectious diseases in China and is increasingly gaining recognition for its potential role in managing brucellosis (159, 221). One of the most well-documented examples of TCM's therapeutic potential is artemisinin, derived from *Artemisia annua*, which has been adopted globally as a first-line treatment for malaria (221). Moreover, herbal formulations have demonstrated efficacy in alleviating symptoms and reducing hospital stays in patients with viral infections such as SARS and COVID-19 owing to their immunomodulatory and anti-inflammatory effects (222–224).

In the context of brucellosis, TCM is often employed as an adjunct to antibiotic therapy to enhance treatment outcomes, minimize side effects, and reduce the risk of antimicrobial resistance (225, 226). Zhang et al. (227) identified ten medicinal herbs that are frequently used to treat brucellosis: *Gan Cao* (GC), *Dang Gui* (DG), *Fu Ling* (FL), *Chen Pi* (CP), *Bai Shao* (BS), *Chuan Xiong* (CX), *Bai Zhu* (BZ), *Huang Qi* (HQ), *Dang Shen* (DS), and *Di Huang* (DH). These herbs exhibit a range of pharmacological activities, including analgesic, antioxidant, antibacterial, antiviral, immunoregulatory, and hepatoprotective effects. Notable examples include the antiarthritic activity of GC (228), the antifibrotic action of DG (229), the antidiabetic effects of FL (230), and the antiatherosclerotic and anticancer potential of CX (231). BZ enhances spleen function (232), HQ and DS are known for their nephroprotective effects (233, 234), and DH has demonstrated antitumor properties (235).

The therapeutic effects of TCM are attributed to bioactive compounds within the plants, some of which may have direct activity against *Brucella* spp. (236). Wen et al. (237) evaluated the antibacterial properties of ten ethanol extracts from herbs used in Malaysian Chinese medicine against *B. melitensis*. Using disc diffusion assays, four extracts—*Coptis chinensis, Radix paeoniae rubra, Galla chinensis*, and *Cortex phellodendrin*—showed inhibitory activity, with minimum inhibitory concentrations (MICs) ranging from 3.75 to 30 mg/ml. These findings suggest the potential utility of these herbs as prophylactic or therapeutic agents against brucellosis.

*Coptis chinensis* (Huanglian) is particularly noteworthy for its antimicrobial potency. Kim et al. (238) demonstrated that ethanol extracts of *C. chinensis* and its major constituents—berberine and palmatine—exhibited inhibitory activity against *B. abortus* at concentrations of 1,000 μg/ml and 100 μg/ml, respectively. However, the extracts and isolated compounds had limited effects on the intracellular survival and replication of *Brucella* within RAW 264.7 macrophages, indicating that while they are bacteriostatic, their intracellular efficacy may be restricted. Further exploration by Xuan et al. (239) highlighted the antiadhesive potential of emodin, an anthraquinone compound derived from traditional herbs. Emodin significantly reduced *B. abortus* entry into macrophages and decreased bacterial adhesion at the highest non-cytotoxic dose. These effects were associated with reduced ERK1/2 phosphorylation and F-actin polymerization, suggesting disruption of host-pathogen interactions. Although emodin does not inhibit *Brucella* growth directly, its ability to modulate host cell signaling implies a promising adjunctive role.

The therapeutic potential of *Caryopteris mongolica* root extract, which is used in traditional Mongolian medicine, was evaluated *in vivo* by Tsevelmaa et al. (240) BALB/c mice were treated for 21 days with doxycycline (2 mg/day), a combination of doxycycline (1 mg/day) and *C. mongolica* extract (20 mg/day), or the extract alone. Compared with the controls, all the treatment groups presented significant reductions in splenic bacterial loads, with the combination therapy providing enhanced efficacy. The extract alone reduced the splenic bacterial burden by 1.47 log units, supporting its synergistic potential in brucellosis therapy and the possibility of lowering antibiotic doses to mitigate resistance.

Collectively, Chinese herbal medicines, including compound formulations and monomeric constituents, display diverse antibacterial mechanisms (241). These include limiting bacterial gene expression, modulating immune responses, and reducing the release of proinflammatory mediators. Ongoing research into TCM-derived compounds is driving the development of novel therapeutics, particularly those against antibiotic-resistant pathogens (242). By elucidating the molecular interactions between herbal bioactives and bacterial or host targets, TCM offers a complementary strategy to conventional antibiotic therapy—one that may help curtail resistance, enhance treatment efficacy, and provide alternative or adjunctive options in brucellosis management.

## 6 Vaccination

Vaccination remains a cornerstone in the control and prevention of animal brucellosis, with significant implications for public health (243–246). Live attenuated vaccines such as *B. abortus* S19 and RB51 for cattle and *B. melitensis* Rev.1 for small ruminants are widely employed in various countries. However, these vaccines pose notable challenges, including the risk of accidental human infection and adverse effects in animals, particularly abortion in pregnant livestock (245, 247–249). Furthermore, standard serological tests used to detect *Brucella* infections cannot reliably differentiate between vaccine and field strains or detect antibodies specific to RB51 (250). In contrast, molecular methods such as PCR provide higher specificity and can distinguish between vaccine and wild-type strains (147).

At present, no vaccines are approved for human use. The potential for severe side effects makes current animal vaccines unsuitable for human application (251). This has spurred research into safer and more effective human vaccine candidates. Despite their importance in reducing zoonotic transmission, current animal vaccines have limitations, including short-term efficacy, hypersensitivity reactions, and interference with serodiagnosis (252). For example, while the S19 vaccine offers temporary protection, it requires frequent boosters and may elicit hypersensitivity. Other experimental vaccines, such as *B. abortus* 84-C and M-104, are generally safe but can cause severe side effects in some individuals (37, 40).

An emerging strategy for vaccine development involves the use of genetically engineered live vectors derived from nonpathogenic bacteria or viruses that express immunogenic *Brucella* antigens (253). Examples include *Lactococcus lactis* (254), *Escherichia coli* (255), *Salmonella enterica* (256), and Semliki Forest virus (257). These vectors have been shown to infect a variety of cell types and express antigens intracellularly, promoting robust immune responses (253). One such example is the Flu-BA vaccine, which employs recombinant influenza viruses (H5N1 as the prime and H1N1 as the booster) to deliver OMP 16 and ribosomal protein L7/L12, with the aim of protecting cattle against *B. abortus* (258, 259).

Subunit vaccines, which are composed of purified antigens such as Omp31, BP26, and L7/L12 or outer membrane vesicles, offer a safer alternative to live vaccines. These compounds have shown immunogenicity in murine models but often require strong adjuvants and multiple doses to achieve protective immunity (175). Among the subunit approaches, DNA vaccines have garnered significant interest. These vaccines encode antigenic components of *Brucella* and stimulate both humoral and cellular responses. They are inherently safe, contain CpG motifs for immune stimulation, and do not require complex storage conditions (76, 260).

DNA vaccines for brucellosis frequently target genes essential for *Brucella*'s intracellular survival and virulence, including bvrR/bvrS (261), Cu-Zn superoxide dismutase (262), ribosomal L7/L12, *Brucella* lumazine synthase (BLS) (76), Omp31 and Omp25 (263), BCSP31 (264), SP41 (265), and ribosomal protein L9 (266). These antigens have been shown to elicit protective immune responses in animal models challenged with virulent strains such as *B. abortus* S19 and 2308 and *B. melitensis* 16 M and Rev.1 (264, 265). DNA vaccine development holds promise for overcoming limitations associated with current live attenuated vaccines (261, 264, 267). However, despite their potential, DNA vaccines generally elicit weaker immune responses in humans than in animal models—particularly in mice—underscoring the need for improved delivery systems and optimized codon usage to increase their efficacy (252).

NP-based vaccine delivery has shown promise in enhancing immune responses. In animal models, NPs containing *Brucella* antigens effectively elicit IgM, IgA, and IgG responses and promote T-helper 1 (Th1) and T-helper 17 (Th17) cell-mediated immunity (3, 268). However, NP-based vaccines are not yet recommended for human use because of concerns about antigen loading efficiency, immune activation capacity, and potential toxicity or disease transmission risks (269, 270). Strategies that integrate LPS and oligosaccharide antigens into poly(lactic-co-glycolic acid) (PLGA) NPs have demonstrated enhanced antibody production, offering significant protective benefits in animal models (271).

The use of recombinant peptides in vaccine design represents another innovative approach to brucellosis prevention. These peptides provide a safer and more targeted alternative to traditional vaccines, avoiding the risks of abortion and diagnostic interference associated with live attenuated vaccines such as Rev.1 (272). One promising candidate, rBtuB-Hia-FlgK, has demonstrated the capacity to enhance CD4+ and CD8+ T-cell responses to *Brucella* antigens (273). Compared with attenuated vaccines, recombinant peptide vaccines could achieve protective efficacy while offering improved safety profiles for use in both livestock and humans.

The successful development of a human brucellosis vaccine necessitates a comprehensive understanding of *Brucella* pathogenicity and host immune interactions. Although DNA vaccines are particularly suited for inducing cell-mediated immunity, they must overcome limitations related to immunogenicity in humans (274). Innovative strategies such as codon optimization, advanced delivery systems, and adjuvant formulations are being explored to improve their efficacy (275). Additional techniques—including transposon mutagenesis, the creation of green fluorescent protein-tagged strains, gene knockouts, and high-throughput bacterial imaging—are being employed to identify and evaluate novel vaccine targets (276).

Ultimately, vaccine candidates must demonstrate efficacy in preclinical models (e.g., mice and non-human primates) and undergo rigorous safety and immunogenicity testing before they can be approved for human use. Although clinical trials in humans remain challenging, the integration of genomics, immunology, and nanotechnology is paving the way for next-generation brucellosis vaccines that could be safer, more effective, and more broadly applicable (251).

## 7 Socioeconomic burden associated with brucellosis

Brucellosis imposes a significant socioeconomic burden worldwide, particularly in regions where livestock farming is a primary source of income. In animals, the disease leads to direct economic losses through decreased productivity, reproductive failure, abortion, and reductions in milk and meat yields (277). These losses are further compounded by expenses related to control strategies, including diagnostic testing, veterinary care, vaccination programs, culling of infected animals, and the implementation of stringent biosecurity measures (278, 279). In addition to animal health, brucellosis represents a major public health concern. Infected individuals often experience non-specific but debilitating symptoms such as fever, fatigue, arthralgia, and prolonged illness (280), which can significantly impair work capacity and reduce economic productivity. The associated costs of medical diagnostics, long-term antibiotic therapy, and follow-up care place financial strain on both affected individuals and healthcare systems (281).

The economic impact extends to international trade and food security. The presence of brucellosis in livestock populations limits market access for animals and animal-derived products (282). Several countries, including Australia, the United States, and New Zealand, have enacted strict regulations regarding the import and export of livestock to prevent the spread of infectious diseases (283). Consequently, brucellosis outbreaks can result in trade restrictions, disrupting the global market for cattle and related commodities (284, 285). Preventive measures are critical to curbing transmission, especially given the zoonotic potential of brucellosis through the consumption of unpasteurized dairy products and undercooked meat. Ensuring food safety through proper hygiene practices, including pasteurization and effective disease surveillance systems, is essential (286). Compliance with international food safety standards not only mitigates the spread of brucellosis but also helps maintain public confidence in food production systems (287).

In addition to affecting humans and domestic animals, brucellosis poses ecological risks by impacting wildlife populations, particularly in regions where wild and domesticated animals share habitats. Wildlife species such as elk and bison can act as reservoirs for the disease, perpetuating transmission cycles and complicating eradication efforts (288). These infections can alter wildlife population dynamics by reducing reproductive success and increasing mortality rates (289). In summary, brucellosis is a multifaceted disease with profound socioeconomic consequences. Addressing these challenges requires a holistic, One Health approach involving coordinated efforts across veterinary, medical, environmental, and regulatory sectors to effectively control and mitigate its widespread impact.

## 8 Challenges and future directions

Brucellosis remains a critical public health and veterinary concern globally, particularly in regions where animal husbandry is intensive and healthcare infrastructure is limited. The disease is notoriously difficult to diagnose owing to its non-specific clinical presentation, which often mimics other febrile or inflammatory illnesses, leading to delayed or misdiagnosed cases. Such diagnostic ambiguity contributes to prolonged illness, increased morbidity, and the potential for ongoing transmission (290). The zoonotic nature of brucellosis further complicates control efforts, as transmission can occur through the consumption of unpasteurized dairy products, direct contact with infected animals, or inhalation of contaminated aerosols—placing high-risk groups such as farmers, veterinarians, abattoir workers, and consumers at continual risk (277, 291, 292).

In endemic regions, disease control is hindered by inadequate healthcare infrastructure, insufficient veterinary coverage, a lack of public awareness, and poor surveillance systems (293, 294). The growing issue of antibiotic resistance in *Brucella* spp. adds another layer of complexity, threatening the efficacy of current therapeutic regimens and highlighting the urgent need for new antimicrobial strategies (42, 295). Addressing these multifaceted challenges necessitates a comprehensive and collaborative One Health approach that integrates human, animal, and environmental health. Priorities should include improved disease surveillance, public health education, and expanded access to healthcare and veterinary services, particularly in resource-limited settings.

Historic eradication programs offer valuable insights for guiding future brucellosis control strategies. In the European Union, coordinated efforts that combined mass vaccination, test-and-slaughter protocols, strict animal movement controls, and mandatory dairy pasteurization enabled many member states to secure official brucellosis-free status (296). In the United States, the longstanding National Brucellosis Eradication Program has virtually eliminated bovine brucellosis, with occasional spillover cases persisting only in wildlife reservoirs such as in the Greater Yellowstone Area (297, 298). New Zealand offers another exemplar: a national campaign initiated in the 1970s, featuring compulsory herd testing, slaughter of reactors, movement restrictions, and farmer compensation, culminated in the country being officially declared brucellosis-free (299–301). These programs demonstrate that elimination is attainable when surveillance and vaccination are coupled with compensation frameworks, rigorous enforcement, and sustained political engagement. Embedding these successful models within a modern One Health framework is key to adapting eradication strategies to the socioeconomic and infrastructural challenges of endemic regions.

Future research must delve deeper into the molecular mechanisms underlying *Brucella* pathogenesis, particularly *Brucella*'s ability to evade host immune responses by modulating key cellular processes such as autophagy and apoptosis (302). Omics technologies, including genomics and proteomics, hold promise for identifying novel virulence factors and vaccine candidates that could inform next-generation immunization strategies (303, 304). The emergence of antimicrobial resistance underscores the need for innovative therapeutics, including the use of monoclonal antibodies, host-directed therapies, and repurposed drugs with enhanced activity against *Brucella* (305). Moreover, combining conventional antibiotics with emerging modalities such as bacteriophage therapy may provide synergistic effects and improve clinical outcomes (306).

Public health and veterinary professionals play vital roles in advancing brucellosis control through education, early detection, and disease reporting. The application of the One Health concept is pivotal for successful management, encompassing livestock immunization, animal hygiene, wildlife monitoring, and intersectoral collaboration (307, 308). During outbreaks, rapid interventions such as livestock quarantine and movement restrictions are essential to limit disease spread. In healthcare settings, clinicians must maintain a high index of suspicion for brucellosis in patients with compatible symptoms and relevant exposure histories (309–311). Enhanced food safety practices—including pasteurization, safe processing of dairy and meat products, and rigorous monitoring systems—are indispensable for reducing transmission risk. Historical accounts, such as the restriction of unpasteurized milk during wartime to prevent brucellosis among British soldiers, underscore the importance of stringent food safety regulations (312). Laboratory and veterinary personnel working with *Brucella* cultures should receive adequate biosafety training and utilize personal protective equipment to minimize occupational risk.

Although no vaccine is currently approved for human use, significant progress has been made in the development of novel animal vaccines, including vector-based, recombinant, DNA, and subunit vaccines. These strategies aim to reduce disease incidence in animal reservoirs and indirectly curb zoonotic transmission. Continued research and investment are needed to optimize these candidates for broader application and eventual translation into human use.

## 9 Conclusions

Brucellosis remains a persistent global health threat at the crossroads of human, animal, and environmental health. Its chronic nature, diagnostic ambiguity, and intracellular persistence—driven by immune-evasive mechanisms such as low-immunogenic LPS and specialized adhesins—complicate detection and treatment, particularly in resource-limited settings. While molecular diagnostics and novel biosensors show promise, conventional serology still dominates in endemic areas despite its limitations. Prolonged antibiotic regimens face challenges such as high relapse rates and increasing resistance. Emerging therapies, including nanotechnology-based delivery systems, host-targeted approaches, and traditional phytomedicines, offer promising alternatives. Preventive efforts have largely focused on animal vaccination, yet the lack of a human vaccine remains a significant gap. Advances in DNA, subunit, and vector-based vaccines show potential but require further development and validation. Tackling brucellosis demands a One Health approach—integrating medical, veterinary, and environmental strategies. Strengthening diagnostics, expanding access to care, and fostering cross-sector collaboration are essential for reducing the global burden. Continued innovation and coordinated policy efforts are critical to transforming scientific progress into sustainable public health solutions.
